# Isolation of Ancestral Sylvatic Dengue Virus Type 1, Malaysia

**DOI:** 10.3201/eid1611.100721

**Published:** 2010-11

**Authors:** Boon-Teong Teoh, Sing-Sin Sam, Juraina Abd-Jamil, Sazaly AbuBakar

**Affiliations:** Author affiliation: University of Malaya, Kuala Lumpur, Malaysia

**Keywords:** Ancestral, dengue virus, viruses, Malaysia, monkey, mosquito, phylogenetic analysis, sylvatic, dispatch

## Abstract

Ancestral sylvatic dengue virus type 1, which was isolated from a monkey in 1972, was isolated from a patient with dengue fever in Malaysia. The virus is neutralized by serum of patients with endemic DENV-1 infection. Rare isolation of this virus suggests a limited spillover infection from an otherwise restricted sylvatic cycle.

Dengue virus (DENV) is a mosquito-borne pathogen maintained in sylvatic (nonhuman primate/sylvatic mosquitoes) and endemic (human/urban/peridomestic mosquitoes) cycles. The endemic form of DENV poses a serious health threat to >100 million persons living in dengue-endemic regions (*1*). The endemic form of DENV may have originated from adaptation of sylvatic DENV to either peridomestic/urban mosquitoes or nonhuman primate hosts 100–1,500 years ago (*2*).

All 4 DENV genotypes are thought to have independently evolved from a sylvatic ancestral lineage, perhaps in Malaysia (*2*). However, only sylvatic DENV-1, DENV-2, and DENV-4 have been isolated, and monkey seroconversion against DENV-1, DENV-2, and DENV-3 has been demonstrated (*3*). Incidences of spillover infection involving sylvatic DENV-2 have been reported, but mainly in West Africa.

Sylvatic dengue may still be endemic to West Africa, especially in areas with dense human habitation near forest areas (*4*,*5*). Sporadic reports of sylvatic dengue may be the result of low incidence of severe forms of this disease in these regions. In contrast, infection with sylvatic dengue is rare in other parts of the world, especially in Southeast Asia where dengue is hyperendemic. Sylvatic DENVs (DENV-1, DENV-2, and DENV-4) were last isolated from monkeys in Malaysia in the 1970s (*3*).

During 2004–2007, a dramatic increase occurred in the number of suspected dengue cases in Malaysia; 155,424 cases and 358 deaths were reported (*6*). DENV-1 was the predominant virus isolated and accounted for 68% of all DENVs isolated. This outbreak represented a third cycle that involved DENV-1 in Malaysia since the 1960s (*7*). We report isolation of DENV-1 that shared >97% genome sequence similarity to an ancestral DENV-1 isolated from a sentinel monkey in Malaysia in 1972 (*3*).

## The Study

At least 442 DENV-1 isolates from the 2004–2007 dengue outbreak were obtained from the Diagnostic Virology Repository at the University of Malaya Medical Centre. Viral RNA was extracted from infected cell culture supernatants, and a 1-step reverse transcription–PCR amplification of the DENV-1 envelope gene was performed by using amplification primers (*8*). Amplified fragments were purified and sequenced by Macrogen Inc. (Seoul, South Korea).

DENV-1 genome sequences from study isolates and those obtained from GenBank (Table 1) were used to construct phylogenetic trees. Maximum clade credibility was inferred by using the Bayesian Markov chain Monte Carlo method implemented in BEAST version 1.5.2 (*9*). For simplicity, only 10 new DENV-1 sequences from the study and 47 from GenBank were analyzed.

**Table 1:** Sylvatic and endemic dengue virus isolates used in the study, Malaysia

Isolate*	Year isolated	GenBank accession no.
D1.Malaysia.36046/05	2005	FN825674
D1.Malaysia.32581/04	2004	FR666923
D1.Malaysia.32858/04	2004	FR666921
D1. Malaysia.33087/04	2004	FR666922
D1. Malaysia.33370/04	2004	FR666923
D1.Malaysia.36000/05	2005	FR666924
D1.Malaysia.36139/05	2005	FR666925
D1.Malaysia.32694/04	2004	FR666926
D1.Malaysia.35765/05	2005	FR666927
D1.Malaysia.35845/05	2005	FR666928

*Isolate D1.Malaysia.36046/05 is a sylvatic type. All other isolates are endemic types.

Phylogenetic trees showed 6 distinct DENV-1 subgenotypes: 3 ancestral subgenotypes (Hawaii/Japan, 1940s; Thailand, 1960s; and Malaysia, 1972) and 3 major endemic subgenotypes (SI, SII, and SIII), which is consistent with reported findings (*8*). An isolate identified as D1.Malaysia.36046/05 grouped with isolate P72_1244, a sylvatic DENV-1 reportedly isolated from a sentinel monkey in Malaysia in 1972. Virus envelope gene sequence shared >97% nt sequence similarities and >99% aa sequence similarities. There was only 1 aa difference at position 55, from valine in P72_1244 to isoleucine in D1.Malaysia.36046/05.

Focus-reduction neutralization tests (FRNTs) were performed by using the D1.Malaysia.36046/05 isolate. Serum samples from patients with primary dengue caused by DENV-1 SI and SII (Figure) were pooled and used in FRNTs as described (*10*). Neutralizing antibody titer was defined as the reciprocal of the highest serum dilution that reduced viral foci by 50% (FRNT_50_). FRNT results after adjustment of the titer to that of respective isolates showed that the D1.Malaysia.36046/05 virus is neutralized by serum from patients with DENV-1 SI infections (FRNT_50_ = 320) and samples from patients with DENV-1 SII infections (FRNT_50_ = 80) (Table 2).

**Figure:**
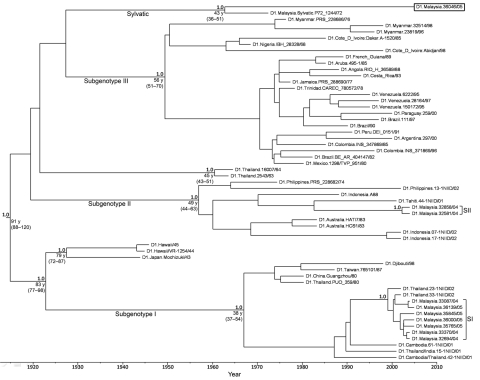
Maximum clade credibility tree of complete envelope genes of dengue virus type 1 (DENV-1) isolates. Horizontal branches are drawn to a scale of estimated year of divergence. Coalescent times with 95% highest posterior density values (ranges in parentheses) and posterior probability values (all 1.0) of key nodes are shown. Patient convalescent-phase serum samples used for neutralization assays from which virus was isolated are indicated at the end of branches according to their virus groups. Box indicates sylvatic DENV-1 isolated in the study. New sequences were used to create the phylogenetic tree are as in Table 1. SII, subgenotype II, SI, subgenotype I.

**Table 2:** Serum neutralization of ancestral sylvatic dengue virus isolate D1/Malaysia/36046/05, Malaysia*

Serum group*	Neutralizing antibody titer†
Mock	0
Virus	0
Medium	0
SI	320
SII	80

*Mock, controls treated with serum from healthy (no dengue infection) donors; virus, virus plus diluent; medium, serum and diluent without virus; SI, subgenotype I; SII, subgenotype II. †Reciprocal of the highest serum dilution that reduced viral foci by 50% (50% focus-reduction neutralization test). Serum from patients infected with sylvatic virus was not available. Virus was treated with serum from patients infected with primary dengue virus type 1 SI or SII.

Laboratory and clinical records showed that D1.Malaysia.36046/05 virus was isolated from a patient who had headache, body ache, chills, rigors, and abdominal pain for 3 days and sought treatment at the University of Malaya Medical Centre. The patient was treated as an outpatient and suspected of having dengue fever. Serologic results for dengue immunoglobulin M were negative. D1.Malaysia.36046/05 was isolated and identified initially as DENV-1 by using immunofluorescent antibody staining. The patient did not return for subsequent follow-up, and efforts to locate the patient were unsuccessful. The most recent address of the patient was within a high population–density area of Kuala Lumpur. Additional sequencing of other DENV-1 isolates from the 2004–2007 outbreak did not identify any additional D1.Malaysia.36046/05–like virus.

## Conclusions

Isolation of the ancestral DENV-1 after >30 years suggests that a mosquito–host transmission cycle has maintained this virus. This rare isolation of the virus suggests a restricted transmission cycle. The natural host of the virus cannot be determined conclusively because the only known fact is that the virus was isolated from a patient with dengue fever. The original ancestral DENV-1 isolate P72_1244 was designated as sylvatic because it was isolated from a sentinel monkey in a rural forest (*3*). Its sylvatic origin has recently become uncertain because the virus genome is phylogenetically closer to other endemic DENV-1 lineages (*11*). However, because no virus with high sequence similarities to that of DENV-1 isolate P72_1244 has been isolated over the past 33 years, the virus may have been maintained in a sylvatic cycle through a nonhuman primate/mosquito enzootic cycle.

The estimated sequence evolution rate for D1.Malaysia.36046/05 is 5.20 × 10^–4^ substitutions/site/year. This rate is relatively slower than those for other endemic DENV-1 isolates used in this study (5.67 × 10^–4^ to 8.05 × 10^–4^ substitutions/site/year). The much smaller monkey:human population ratio (700,000:28,000,000) (*12*) (http://en.wikipedia.org/wiki/Malaysia) and the more restricted mobility of monkeys could have limited the virus genome sequence divergence, leading to conservation of the sylvatic virus genome sequence.

The absence of the virus from the endemic urban cycle over the past 33 years could have been caused by its inability to overcome population herd immunity after exposure to endemic DENV-1. Efficient neutralization of virus by serum from patients infected with DENV-1 SI and SII supports this possibility (*13*). Conversely, the virus may not be highly transmissible by peridomestic mosquitoes (*14*) and may be confined to the enzootic forest cycle. Therefore, isolation of the ancestral virus from a person living in Kuala Lumpur is most likely the result of a stochastic spillover event after contact with infected forest-dwelling mosquitoes.

We report isolation of an ancestral sylvatic DENV-1 from an infected person. Available evidence does not support endemic presence of the virus in an urban dengue cycle. However, a sylvatic cycle needs to be considered in any future dengue vaccination initiatives.
